# Procalcitonin Increase Is Associated with the Development of Critical Care-Acquired Infections in COVID-19 ARDS

**DOI:** 10.3390/antibiotics10111425

**Published:** 2021-11-22

**Authors:** Owen Richards, Philip Pallmann, Charles King, Yusuf Cheema, Charlotte Killick, Emma Thomas-Jones, Jessica Harris, Catherine Bailey, Tamas Szakmany

**Affiliations:** 1 School of Medicine, Cardiff University, Cardiff CF14 4XN, UK; richardso3@cardiff.ac.uk (O.R.); kingc20@cardiff.ac.uk (C.K.); cheemaya@cardiff.ac.uk (Y.C.); killickcj@cardiff.ac.uk (C.K.); 2 Centre for Trials Research, Cardiff University, Cardiff CF14 4XN, UK; pallmannp@cardiff.ac.uk (P.P.); thomas-jonese@cardiff.ac.uk (E.T.-J.); 3 Department of Clinical Biochemistry, Grange University Hospital, Aneurin Bevan University Health Board, Cwmbran NP44 2XJ, UK; jessica.harris2@wales.nhs.uk (J.H.); catherine.bailey2@wales.nhs.uk (C.B.); 4 Department of Anaesthesia, Intensive Care and Pain Medicine, Cardiff University, Cardiff CF14 4XN, UK; 5 Critical Care Directorate, Grange University Hospital, Aneurin Bevan University Health Board, Cwmbran NP44 2XJ, UK

**Keywords:** COVID-19, procalcitonin, C-reactive protein, secondary infection

## Abstract

Secondary bacterial infection in COVID-19 patients is associated with increased mortality and disproportionately affects critically ill patients. This single-centre retrospective observational study investigates the comparative efficacy of change in procalcitonin (PCT) and other commonly available biomarkers in revealing or predicting microbiologically proven secondary infection in critical COVID-19 patients. Adult patients admitted to an intensive care unit (ICU) with confirmed SARS-CoV-2 infection between 9 March 2020 and 5 June 2020 were recruited to the study. For daily biomarker and secondary infection, laboratory-confirmed bloodstream infection (LCBI) and ventilator-associated pneumonia/tracheobronchitis (VAP/VAT) data were collected. We observed a PCT rise in 53 (81.5%) of the patients, a C-reactive protein (CRP) rise in 55 (84.6%) and a white blood cell count (WBC) rise in 61 (93.8%). Secondary infection was confirmed in 33 (50.8%) of the patients. A PCT rise was present in 97.0% of patients with at least one confirmed VAP/VAT and/or LCBI event. CRP and WBC rises occurred in 93.9% and 97.0% of patients with confirmed VAP/VAT and/or LCBI, respectively. Logistic regression analysis found that, when including all biomarkers in the same model, there was a significant association between PCT rise and the occurrence of LCBI and/or VAP/VAT (OR = 14.86 95%CI: 2.20, 342.53; *p* = 0.021). Conversely, no statistically significant relationship was found between either a CRP rise (*p* = 0.167) or a WBC rise (*p* = 0.855) and the occurrence of VAP/VAT and/or LCBI. These findings provide a promising insight into the usefulness of PCT measurement in predicting the emergence of secondary bacterial infection in ICU.

## 1. Introduction

Coronavirus disease 2019 (COVID-19) has been implicated in over four million deaths worldwide since its emergence in late 2019 [1]. Caused by infection with the SARS-CoV-2 virus, it is a disease with a highly variable presentation, ranging from asymptomatic infection to severe pneumonia, acute respiratory distress syndrome (ARDS), multiple organ failure and death [2]. Concurrent bacterial infection in a critically ill patient can compound this complexity; therefore, early and accurate recognition of secondary pathogens is imperative in effectively managing an already unpredictable disease.

Procalcitonin (PCT) is a biomarker with efficacy in differentiating bacterial from viral pneumonia [3], and serum levels correlate with infection severity [4]. Secondary bacterial infection in COVID-19 patients is associated with increased mortality [5,6] and disproportionately affects critically ill patients [7,8]. Consistent monitoring of PCT levels in critical care may provide a crucial insight in detecting or predicting co-infection, indicating the magnitude of the bacterial burden and guiding treatment. Whilst PCT is utilised in ICUs across the UK, its use has been inconsistent and unevidenced [9]. Furthermore, no data currently demonstrate the value of dynamic PCT changes in the presence or absence of confirmed secondary infection in COVID-19 patients [10], illustrating the urgent need for further research to allow for the formulation of a usable algorithm.

### Aims and Objectives

Our study aims to investigate the comparative efficacy of PCT and other commonly available biomarkers in revealing or predicting microbiologically proven secondary bacterial infection in an ICU COVID-19 patient.

## 2. Results

Sixty-five patients with a positive SARS-CoV-2 PCR test were admitted to an ICU between the specified observation dates and included in the analysis. As per our admission policy to the ICU, all patients were mechanically ventilated and fulfilled the Berlin definition of ARDS [11]. Baseline demographics, mortality rates and Sequential Organ Failure Assessment (SOFA) score information are detailed in Table 1.

We observed a PCT rise in 53 (81.5%) of the patients, a CRP rise in 55 (84.6%) and a white blood cell count (WBC) rise in 61 (93.8%). The patient level data on the kinetics of the three inflammatory markers is provided in Supplementary Figures S1–S3.

**Figure 1:**
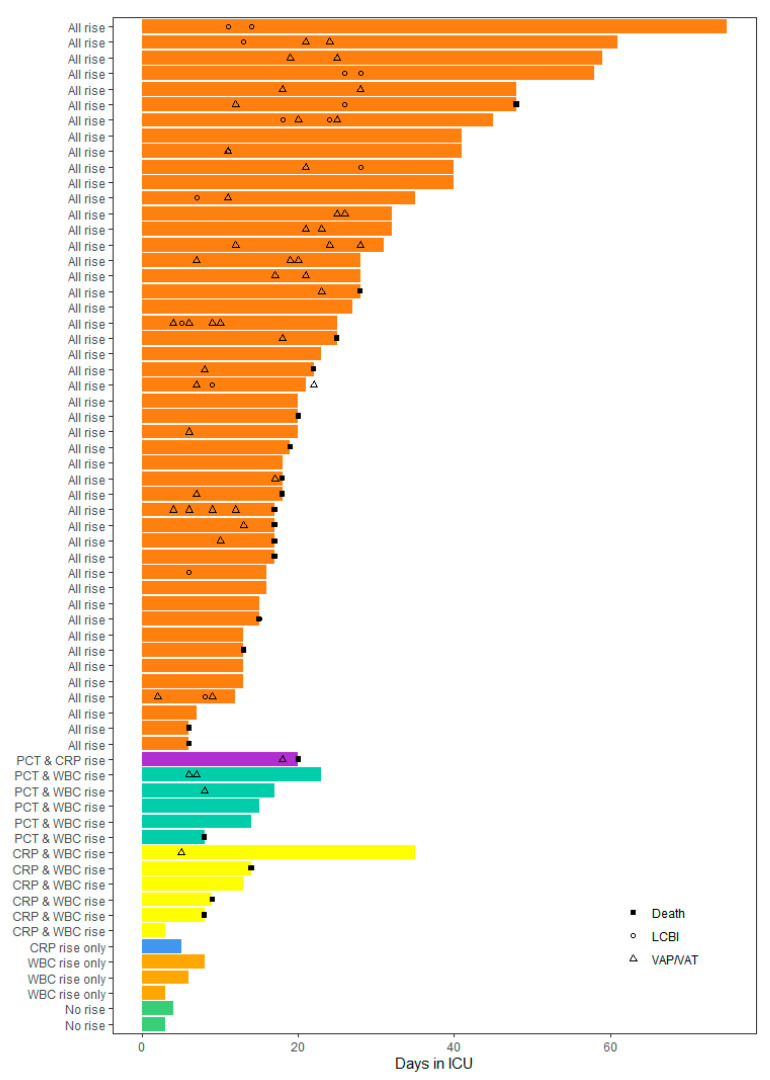
Changes in procalcitonin (PCT), C-reactive protein (CRP) and white blood cell count (WBC) with respect to the timing of confirmed ventilator-associated pneumonia/tracheobronchitis (VAP/VAT) (∆), laboratory-confirmed bloodstream infection (LCBI) (°) and death (■) on the ICU.

Secondary infection was confirmed in 33 (50.8%) of the patients. Ventilator-associated pneumonia/tracheobronchitis (VAP/VAT) was the most common ICU-acquired infection, occurring in 28/65 (43.1%) patients, of whom 8/65 (12.3%) suffered both VAP/VAT and a laboratory-confirmed bloodstream infection (LCBI) during their ICU stay. Furthermore, 5/65 (7.7%) patients were observed to have LCBI in isolation. It was common that patients experienced multiple episodes of infection, giving an overall incidence of 43.6 secondary infections per 1000 ICU days. Most of the infections could be classed as late, occurring after day 10 of ICU admission. The changes in biomarker levels, with respect to the timing of the ICU-acquired infections, are presented in Figure 1 and in Supplementary Figure S4.

**Figure 2:**
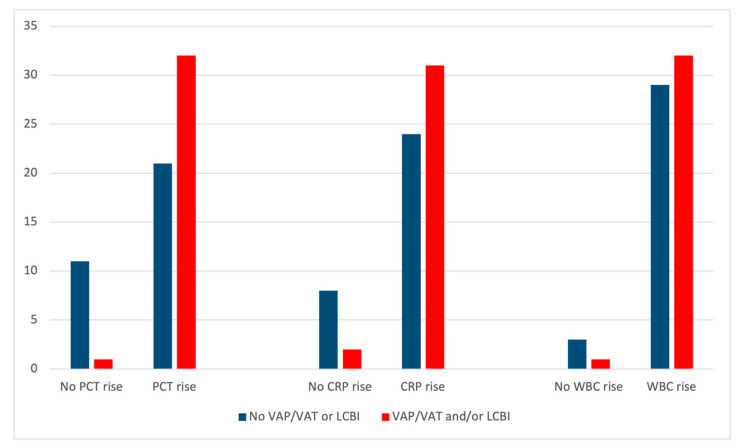
Relative frequencies of patients who experienced a rise in procalcitonin (PCT), C-reactive protein (CRP) and white blood cell count (WBC) in the context of confirmed ventilator-associated pneumonia/tracheobronchitis (VAP/VAT) or laboratory-confirmed bloodstream infection (LCBI).

**Figure 3:**
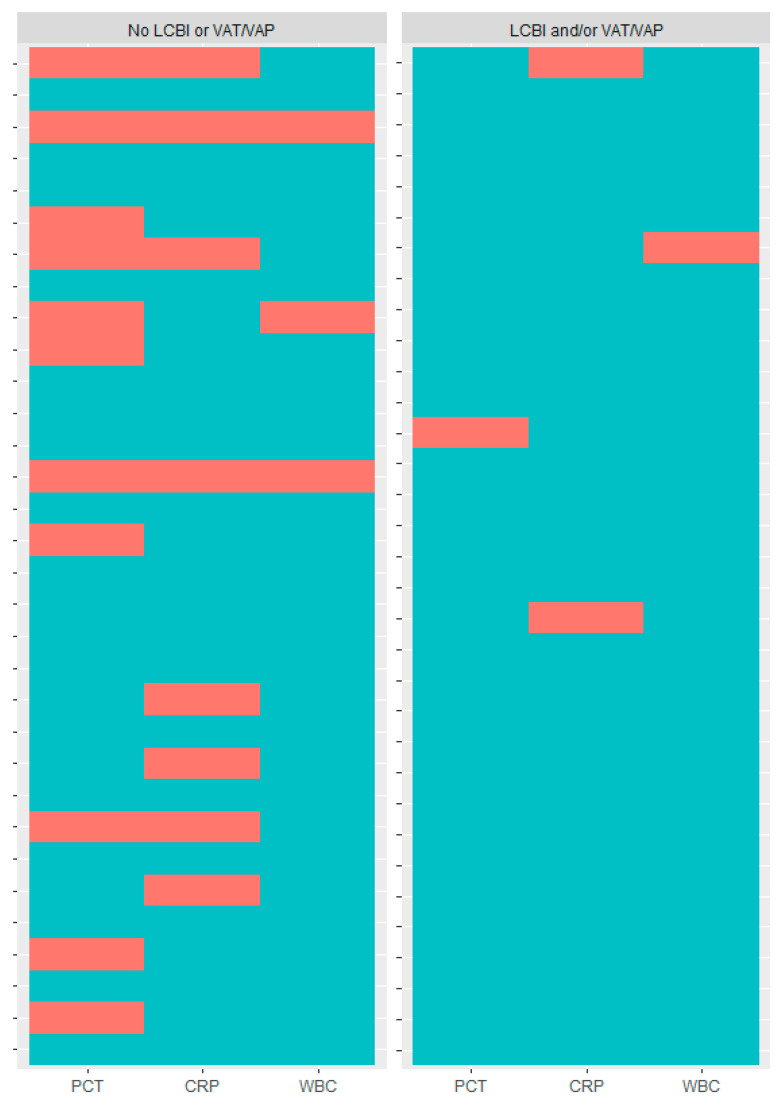
Heatmap of individual patients who experienced a rise in procalcitonin (PCT), C-reactive protein (CRP) and white blood cell count (WBC) in the context of confirmed ventilator-associated pneumonia/tracheobronchitis (VAP/VAT) or laboratory-confirmed bloodstream infection (LCBI). Green = biomarker rise; red = no biomarker rise.

A PCT rise was present in 97.0% of the patients with at least one confirmed VAP/VAT and/or LCBI event. CRP and WBC rises occurred in 93.9% and 97.0% of patients with confirmed VAP/VAT and/or LCBI, respectively. Relative frequencies are detailed in Figure 2 and the individual patient heatmap is presented in Figure 3.

We used logistic regression to estimate the association between PCT rise and the occurrence of LCBI and/or VAP/VAT (at least one event during the ICU stay), whilst controlling for age, sex and the number of comorbidities. When we included all three biomarkers in the model, we found that only the effect of the PCT rise was statistically significant (*p* = 0.021). We developed similar models for each individual marker and found that the effect of the PCT rise was statistically significant (*p* = 0.009). The estimated log odds ratio (OR) associated with PCT rise was 2.85 (95% CI: 1.08, 5.80), corresponding to an OR of 17.25 (95% CI: 2.95, 330.63). A statistically significant relationship was also found between CRP rise and the occurrence of LCBI and/or VAP/VAT (*p* = 0.043). The estimated log OR associated with CRP rise was 1.72 (95% CI: 0.21, 3.70), corresponding to an OR of 5.60 (95% CI: 1.23, 40.38). However, no statistically significant relationship was found between WBC rise and the occurrence of LCBI and/or VAP/VAT (*p* = 0.314). The estimated log OR associated with WBC rise was 1.23 (95% CI: −0.97, 4.31), corresponding to an OR of 3.42 (95% CI: 0.38, 74.61). (Table 2).

The median SOFA score on admission was 10 for patients with (range 6–15) and without (range 3–16) a PCT rise. Overall, mortality among the cohort was 32.3%. Of the 21 patients who died in the ICU, 18 (85.7%) experienced a rise in PCT. In surviving patients, a lower proportion experienced an observed PCT rise (79.5%). However, the association between PCT rise and subsequent death, whilst controlling for age, sex and number of comorbidities, was not statistically significant in a logistic regression analysis (*p* = 0.605).

The length of ICU stay was longer in the patients who experienced a PCT rise, compared to those who did not (Table 1). We used linear regression to estimate the association between PCT rise and length of ICU stay, whilst controlling for age, sex, ethnicity (Caucasian vs. non-Caucasian) and number of comorbidities, and we found that the effect of PCT rise was not statistically significant (*p* = 0.102). Similarly, we found no statistically significant relationship between either CRP rise and length of ICU stay (*p* = 0.300) or WBC rise and length of ICU stay (*p* = 0.638).

## 3. Discussion

This retrospective single-centre cohort study showed that a prespecified rise in PCT by 50%, compared to a previous value at any time point, was significantly associated with the occurrence of secondary infection in ICU patients admitted with critical COVID-19. Conversely, neither CRP nor WBC were shown to have any significant discriminatory associations. This indicates the relative usefulness of serial PCT measurements in the identification of nosocomial bacterial infection and highlights its potential for guiding antimicrobial therapy in COVID-19 ICU patients.

In our study, secondary infection occurred in 51% of patients. This was considerably higher than the levels reported in a living systematic review and meta-analysis of 24 studies (3338 patients), where Langford et al. [8] found documented bacterial infection in only 8.1% (95%CI 2.3–13.8%) of critically ill patients. However, studies of a similar size and setting to our own have reported high levels of secondary infection [12,13]. ICU patients are at heightened risk of nosocomial infection—up to 5 times that of general hospital patients [14]—and major risk factors, such as mechanical ventilation, are especially pertinent in COVID-19 [15]. Indeed, our data showed that VAP/VAT was confirmed in 43% of patients. Similar to our study, a meta-analysis and a large cohort study from Italy, involving an almost identical patient population, recently recorded VAP rates in close to 50% of patients with COVID-19 ARDS [16,17]. As bacterial co-infection increases mortality and prolongs the ICU stay of critical COVID-19 patients [18], early identification of secondary pathogens is crucial.

Our finding, which confirmed that VAP/VAT and/or LCBI were significantly associated with a prespecified rise in PCT but not with a rise in CRP or WBC, suggests that serial PCT measurements could become useful in predicting the emergence of secondary bacterial infection in critical COVID-19 patients. Indeed, this has been supported in other studies, for instance, Pink et al. [13] showed that a PCT < 0.55 ng/mL had a negative predictive value of 93% but that the predictive values of CRP were less robust. Similarly, Van Berkel et al. [12] reported that a PCT level >1.0 ng/mL had a positive predictive value of 93%; however, CRP offered little use when monitoring for bacterial pathogens. The results of these studies provide external validity to our findings and support the use of PCT monitoring in the ICU setting.

In contrast to our findings, Wan et al. [19] reported that despite significantly elevated PCT in patients with more severe diseases, none showed evidence of bacterial infection. In our study, of those patients who did not experience a VAP/VAT or LCBI, 65.6% experienced a PCT rise. This perhaps suggests that a PCT rise is not as a result of bacterial pathogens but a response to systemic inflammatory dysregulation often reported in severe COVID-19 patients [20,21,22], most notably the documented suppression of interferon-γ (IFN-γ), the primary inhibitor of PCT in viral infection [21]. Whilst this is important to consider, Wan et al. [19] derive a significant association between severity and PCT levels based on a PCT threshold of 0.1 ng/mL on admission.

However, relying on specific fixed PCT levels to alert clinicians to bacterial infection and dictate treatment is problematic. Absolute values of inflammatory markers such as PCT and CRP can be readily influenced by disease-modifying agents as we demonstrated earlier [23]. Previously published patient level data derived from the first wave of the pandemic showed that PCT levels can be sporadic and highly variable, with levels elevated even in the absence of confirmed infection [22,23,24]. As such, our study employed a predetermined rise in PCT, utilising trends in serial PCT levels rather than a critical tipping point as an indicator for secondary infection, which could be adopted in clinical practice. PCT kinetics have been successfully used to detect nosocomial infections and to monitor the appropriateness of antimicrobial therapy [25,26,27,28,29,30]. Our definition of PCT rise was based on these previous trials, to provide an easily accessible clinical tool at the bedside.

A multicentre prospective cohort study analysing 48,902 COVID-19 inpatients from 260 hospitals across the UK found that 37% had received antimicrobial therapy in the community prior to admission and 85% received antibiotics as inpatients, with the highest rates reported in critical care [31]. Of their cohort, microbiologically confirmed infections were present in only 1106 (2.3%) patients. Similarly, before the COVID-19 pandemic, we demonstrated significant overuse of antimicrobials, without appropriate investigations in the general ward environment, in patients who had strong clinical suspicion of infection [32,33]. During the first wave of the pandemic, empirical antimicrobials were recommended by NICE without much data available; these recommendations were extrapolated from the H1N1 influenza pandemic [10]. These stark statistics highlight the indiscriminate use of antibiotic therapy in COVID-19 patients and raise concerns about antibiotic resistance, which could present a possible ongoing complication of the COVID-19 pandemic and should be addressed as a matter of urgency. The microbiologically confirmed diagnosis of infection has been shown to represent 50–75% of infections on the ICU, and it is currently unknown which is the best way to approach the diagnosis of culture-negative suspected bacterial infections in the context of COVID-19 ARDS [34]. Biomarkers associated with the development of such infections could be useful tools at the bedside [35].

A survey of antibiotic prescribing conducted in Scotland across 15 hospitals showed that a raised CRP ≥ 100 mg/L was associated with higher odds of antibiotic therapy in COVID-19 patients [36], implying a reliance on CRP in guiding treatment when bacterial infection was suspected. Notably, PCT was not evaluated in the study as it is not routinely used in Scotland. We report that, in a combined model, rises in CRP and WBC were not significantly associated with nosocomial bacterial infection and are therefore of little use in directing antimicrobial treatment strategies. By contrast, the significant association we have shown between a PCT rise and ICU-acquired infection reinforces the potential of using PCT as part of an algorithm to initiate antimicrobial therapy to treat nosocomial infections in the context of a primary viral pathogen-induced acute respiratory failure [37].

The occurrence of healthcare-associated infections is associated with an increased length of stay in COVID-19 pneumonia. On the other hand, secondary infections, especially VAP, are more likely to develop if patients receive prolonged respiratory support [16,38]. We found that ICU length of stay was longer in the patients who experienced a PCT rise, which in turn is associated with secondary infections; however, when controlling for known confounders, such as age, sex and comorbidities, we found no significant associations between biomarker rise and length of stay. This might indicate that secondary infections per se are not major determinants of prolonged ICU stay; however, this would need to be explored in a much larger multicentre dataset. It must be noted that patients in our centre had a prolonged ICU length of stay which fell into to the 75–100% quartile when compared to the rest of the UK [39]. The onset of the first secondary infection in our cohort corresponded to the median length of stay of the rest of the UK and, compared to other international datasets, they would be classified as “late” infections [16,38].

The limitations of our study included the retrospective design and the fact that ~10% of daily laboratory data were missing. Similar missingness has been noted in other retrospective studies [16,38]. Additionally, microbiological investigations were not always performed prior to the initiation of antimicrobial therapy, potentially affecting the detection of a previously established bacterial infection. Our definition of PCT, CRP and WBC rise can be seen as arbitrary. There is no universally accepted value to define the dynamic changes of these biomarkers and, in this context, any such definition has drawbacks. We tried to mitigate this by using delta values from previously published studies [25,26,27,28,29,30]. In our statistical model, we did not specifically control for the timing of the biomarker rise and the development of VAP/VAT or LCBI. Hence, we can only show association, and we cannot make any claims of diagnostic accuracy using these biomarkers. We have previously shown that the diagnostic criteria for these events are very dependent on the framework used, and further analysis is needed to understand if PCT rise could be used as part of this framework [40]. We did not examine the usefulness of any of the markers as stopping guides for unnecessary antimicrobial therapy as we adopted serial PCT measurements as part of standard of care, and we did not have a comparator group, unlike Calderon et al. [30]. The relatively small sample size of our study and the high rates of secondary infection may mean that our results are not generalisable; however, recent data from Italy and Spain involving critical COVID-19 patients suggest that the rate of secondary infection in the ICU might be higher than previously thought [16,18]. We did not examine the potential effects of any disease-modifying treatments, such as corticosteroids, interleukin-6-receptor inhibitors, antivirals or hydroxychloroquine, on secondary infections as we had a small sample size [41]. In the first wave of the COVID-19 pandemic, we only used such therapies as part of a randomised controlled trial [42,43,44,45].

## 4. Materials and Methods

This was a single-centre retrospective observational study conducted in Aneurin Bevan University Health Board in Wales, United Kingdom. The study was reviewed by the local Research and Development Risk Review Group (PICOT: Evaluation of Procalcitonin Measurement in COVID Patients Admitted to ICU, 12 April 2020) and, in accordance with the Health Research Authority’s guidance, was classed as a service evaluation and consent was waived.

Patients admitted to the ICU between 9 March 2020 and 5 June 2020 were screened daily and recruited to the study providing they fulfilled the following inclusion criteria: age ≥ 18 years old; a SARS-CoV-2 infection confirmed by positive RT-PCR.

Data on patient demographics, co-morbidities, daily biomarker and physiological results, SOFA scores on admission and clinical outcomes were collected retrospectively using patient medical notes and laboratory reports. Daily CRP and white blood cell count (WBC) measurements were standard of care in the ICU, and daily PCT measurements were adopted as standard of care on 18 March 2020, providing serial data. Microbiological data identifying pathogens implicated in ICU-acquired secondary infections were also collected as per standard practice. Secondary bacterial infection was classified either as healthcare-acquired laboratory-confirmed bloodstream infection (LCBI) or ventilator-associated pneumonia/tracheobronchitis (VAP/VAT), the definitions of which are detailed in Table 3. Patients were observed from ICU admission until discharge from ICU or death.

### Statistical Analysis

All primary data analyses were carried out according to the prespecified statistical analysis plan (see Supplementary Materials), which was approved before the final database lock. Missing biomarker data were assumed to be missing at random and not replaced. For the primary comparison of patients with vs. without a PCT rise, a logistic regression model was fitted with the occurrence of at least one LCBI or VAP/VAT event as the dependent variable and biomarker rise (yes/no), age, sex and the number of comorbidities as independent variables. Odds ratios were estimated alongside 95% CIs and *p*-values. Similar models were fitted for secondary outcomes, depending on the type of outcome variable (logistic regression for death, linear regression for ICU length of stay), and with different biomarkers (CRP, WBC) as independent variables. Models including all three biomarkers as independent variables were also fitted. Ethnicity (Caucasian vs. non-Caucasian) was planned to be another covariate but had to be removed from the logistic models to be fittable (all Caucasian patients had a biomarker rise). Statistical significance was defined as a *p*-value < 0.05. All statistical analysis was performed using R version 4.1.0.

## 5. Conclusions

In conclusion, our finding, which confirmed that VAP/VAT and/or LCBI was significantly associated with a prespecified rise in PCT, provides a promising insight into the usefulness of PCT measurement in predicting the emergence of secondary bacterial infection in critical care. Secondary infections on the ICU are difficult to treat and associated with worse outcomes. Evaluating biomarker kinetics such as PCT rise might prove useful in directing antimicrobial stewardship.

## Figures and Tables

**Table 1:** Demographics, mortality, SOFA scores and length of stay of ICU COVID-19 patients who experienced a rise in PCT and those who did not.

Variables	No PCT Rise	PCT Rise	Overall
	(*n* = 12)	(*n* = 53)	(*n* = 65)
Age (years)			
Mean (SD)	56.9 (10.6)	55.8 (11.0)	56.0 (10.8)
Median [Min, Max]	56.5 [41.0, 76.0]	57.0 [22.0, 77.0]	57.0 [22.0, 77.0]
Sex			
Female (*n*, %)	4 (33.3%)	18 (34.0%)	22 (33.8%)
Male (*n*, %)	8 (66.7%)	35 (66.0%)	43 (66.2%)
Outcome			
Alive (*n*, %)	9 (75.0%)	35 (66.0%)	44 (67.7%)
Deceased (*n*, %)	3 (25.0%)	18 (34.0%)	21 (32.3%)
Comorbidities			
Diabetes	2 (16.7%)	16 (30.2%)	18 (27.7%)
Hypertension	6 (50%)	22 (41.5%)	28 (43.1%)
Ischaemic heart disease	0 (0%)	3 (5.7%)	3 (4.6%)
COPD	0 (0%)	1 (1.9%)	1 (1.5%)
Asthma	3 (25%)	14 (26.42%)	17 (26.2%)
Chronic renal disease	0 (0%)	2 (3.8%)	2 (3.1%)
Other comorbidities	0 (0%)	10 (18.9%)	10 (15.4%)
Ethnicity			
Black (*n*, %)	1 (3.3%)	1 (2.9%)	2 (3.1%)
Caucasian (*n*, %)	21 (70.0%)	26 (74.3%)	47 (72.3%)
Indian Subcontinent (*n*, %)	4 (13.3%)	4 (11.4%)	8 (12.3%)
Filipino (*n*, %)	3 (10.0%)	2 (5.7%)	5 (7.7%)
Mixed other (*n*, %)	1 (3.3%)	2 (5.7%)	3 (4.6%)
SOFA score on admission			
Mean (SD)	10.6 (4.35)	9.74 (2.74)	9.88 (3.03)
Median [Min, Max]	10.0 [3.00, 16.0]	10.0 [6.00, 15.0]	10.0 [3.00, 16.0]
Length of ICU stay (days)			
Mean (SD)	9.25 (8.93)	25.6 (15.2)	22.6 (15.6)
Median [Min, Max]	7.00 [3.00, 35.0]	20.0 [6.00, 75.0]	18.0 [3.00, 75.0]

SOFA = Sequential Organ Failure Assessment; PCT = procalcitonin; COPD = chronic obstructive pulmonary disease.

**Table 2:** Biomarker data of ICU COVID-19 patients who experienced VAP/VAT and/or LCBI and those who did not.

Change in Biomarker Levels	No VAP/VAT or LCBI	VAP/VAT and/or LCBI	Overall	Multivariable OR (95%CI)	*p*-Value
	(*n* = 32)	(*n* = 33)	(*n* = 65)		
PCT					
No PCT rise	11(34.4%)	1 (3.0%)	12 (18.5%)		
PCT rise	21 (65.6%)	32 (97.0%)	53 (81.5%)	14.86 (2.20, 342.53)	*p* = 0.021
CRP					
No CRP rise	8 (25.0%)	2 (6.1%)	10 (15.4%)		
CRP rise	24 (75.0%)	31 (93.9%)	55 (84.6%)	3.70 (0.62, 30.17)	*p* = 0.167
WBC					
No WBC rise	3 (9.4%)	1 (3.0%)	4 (6.2%)		
WBC rise	29 (90.6%)	32 (97.0%)	61 (93.8%)	0.73 (0.02, 26.48)	*p* = 0.855

Data are *n* (%). *p*-values were calculated by multivariable logistic regression analysis. VAP/VAT = ventilator-associated pneumonia/tracheobronchitis; LCBI = laboratory-confirmed bloodstream infection; PCT = procalcitonin; CRP = C-reactive protein; WBC = white blood cell count; OR = odds ratio; 95%CI = 95% confidence interval.

**Table 3:** Definitions of clinical outcomes.

Outcome	Definition		
Healthcare-acquired laboratory-confirmed bloodstream infection (LCBI)	Patient has a recognised pathogen cultured from one or more blood cultures, and organism cultured from blood is not related to an infection at another site.	OR	Patient has at least one of the following signs or symptoms: fever (>38 °C), chills or hypotension; signs and symptoms and positive laboratory results are not related to an infection at another site and at least one of the following: (a) common skin contaminant (e.g., diphtheroids, Bacillus sp., Propionibacterium sp., coagulase-negative Staphylococci, or Micrococci) is cultured from two or more blood cultures drawn on separate occasions; (b) common skin contaminant is cultured from at least one blood culture from a patient with an intravascular line, and the physician institutes appropriate antimicrobial therapy; (c) positive antigen test on blood or urine (e.g., H. influenzae, S. pneumoniae, N. meningitidis, or Group B Streptococcus).
Ventilator-associated pneumonia/tracheobronchitis (VAP/VAT)	The diagnostic criteria for VAP include: a new infiltrate on chest X-ray associated with at least two of the following: body temperature ≥ 38.5 °C or < 36 °C; leukocyte count ≥ 10 × 10^9^/L or < 1.5 × 10^9^/L; and purulent tracheal aspirate or sputum. In addition, a microbiological confirmation is required for all patients (positive endotracheal aspirate culture ≥ 10^5^ colony-forming units (cfu)/mL or positive bronchoalveolar lavage culture ≥ 10^4^ cfu/mL). VAT is defined using the same criteria as for VAP, except the presence of new or progressive pulmonary infiltrate.		
Procalcitonin (PCT) rise	An increase by at least 50% from previous value at any point in time.		
C-reactive protein (CRP) rise	An increase by at least 50% from previous value at any point in time.		
White blood cell count (WBC) rise	An increase by at least 20% from previous value at any point in time.		

## Data Availability

The data presented in this study are available on request from the corresponding author. The data are not publicly available due to privacy constraints.

## Supplementary Materials

The following are available online at https://www.mdpi.com/article/10.3390/antibiotics10111425/s1, Figure S1: PCT trajectories over time for individual patients (in ng/mL), Figure S2: CRP trajectories over time for individual patients (in mg/L), Figure S3: WBC trajectories over time for individual patients (in 1000/L), Figure S4: Violin plots for days of LCBI and VAP/VAT onset and first biomarker rise.
