# Supplementary material for: Procalcitonin Increase Is Associated with the Development of Critical Care-Acquired Infections in COVID-19 ARDS
Source: Antibiotics (Basel). 2021 Nov 22;10(11):1425. doi: 10.3390/antibiotics10111425 (PMC8615001; doi:10.3390/antibiotics10111425)
Supplement: Supplementary file 1 [file antibiotics-10-01425-s001.zip › antibiotics-1432302-supplementary.pdf]

antibiotics

Article

Procalcitonin Increase Is Associated with the Development of Critical Care-Acquired Infections in COVID-19 ARDS

Owen Richards [1,†], Philip Pallmann [2,†], Charles King [1], Yusuf Cheema [1], Charlotte Killick [1], Emma Thomas-Jones [2], Jessica Harris [3], Catherine Bailey [3] and Tamas Szakmany [4,5,*]

[1] School of Medicine, Cardiff University, Cardiff, CF14 4XN, UK; richardso3@cardiff.ac.uk (O.R.); kingc20@cardiff.ac.uk (C.K.); cheemaya@cardiff.ac.uk (Y.C.); killickcj@cardiff.ac.uk (C.K.)
[2] Centre for Trials Research, Cardiff University, Cardiff, CF14 4XN, UK; pallmannp@cardiff.ac.uk (P.P.); thomas-jonese@cardiff.ac.uk (E.T.-J.)
[3] Department of Clinical Biochemistry, Grange University Hospital, Aneurin Bevan University Health Board, Cwmbran, NP44 2XJ, UK;
jessica.harris2@wales.nhs.uk (J.H.); catherine.bailey2@wales.nhs.uk (C.B.)
[4] Department of Anaesthesia, Intensive Care and Pain Medicine, Cardiff University, Cardiff, CF14 4XN, UK
[5] Critical Care Directorate, Grange University Hospital, Aneurin Bevan University Health Board, Cwmbran, NP44 2XJ, UK
* Correspondence: szakmanyt1@cardiff.ac.uk
† These authors contributed equally to this work

Supplementary Materials

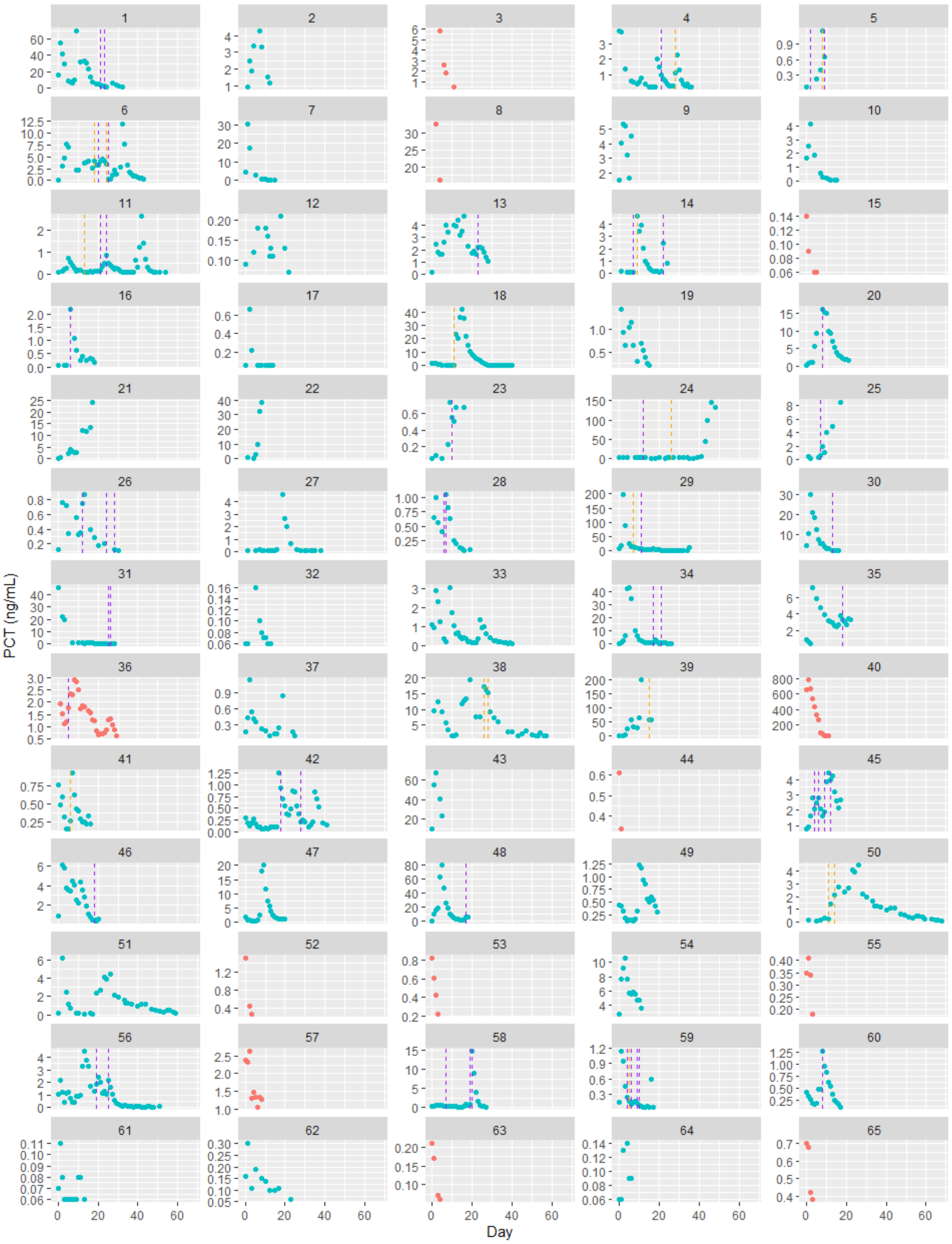

Figure S1. PCT trajectories over time for individual patients (in ng/mL). Green=PCT rise; red=no PCT rise. Purple=VAP/VAT event; orange=LCBI event. NB: y-axis scales are vastly different.

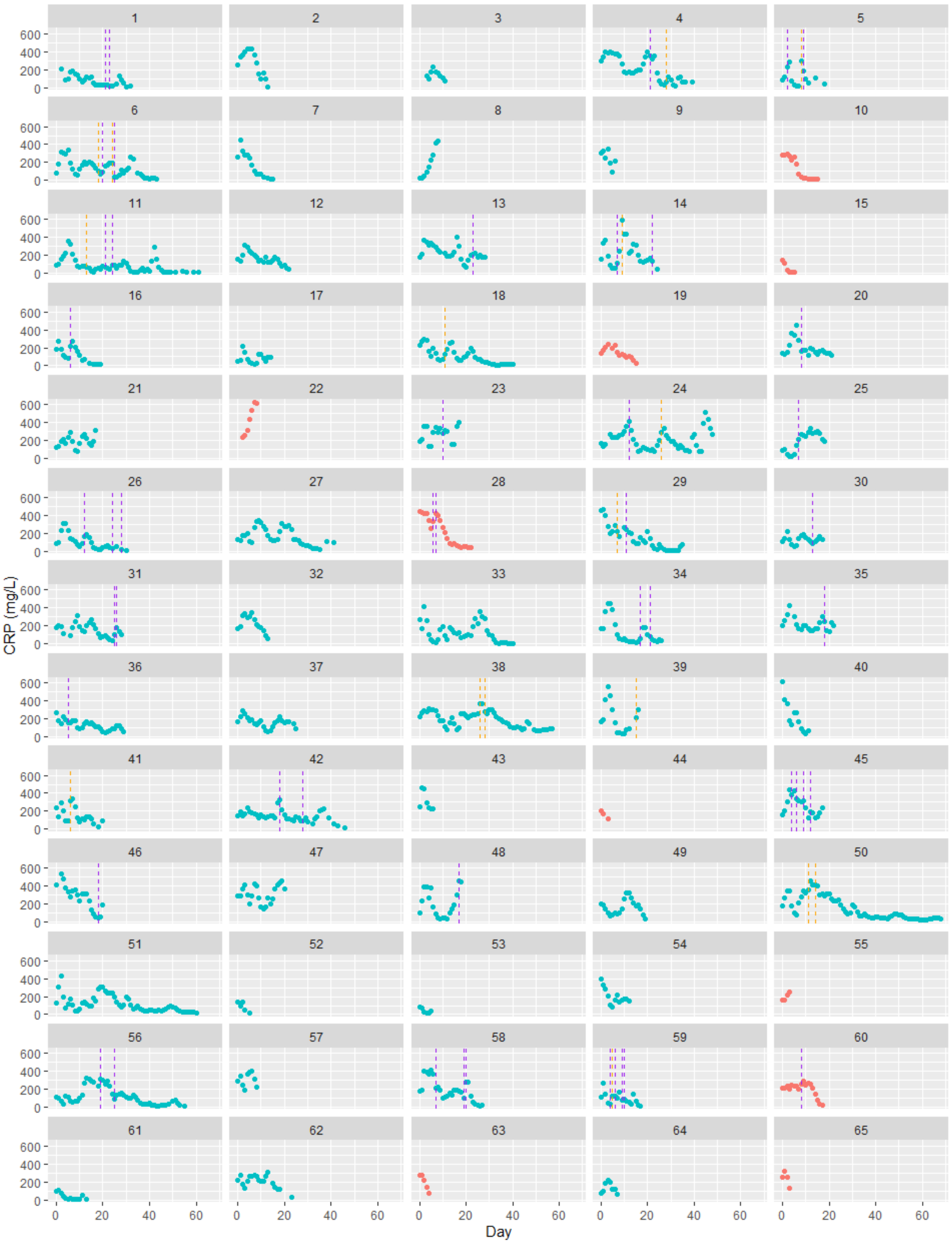

Figure S2. CRP trajectories over time for individual patients (in mg/L). Green=CRP rise; red=no CRP rise. Purple=VAP/VAT event; orange=LCBI event.

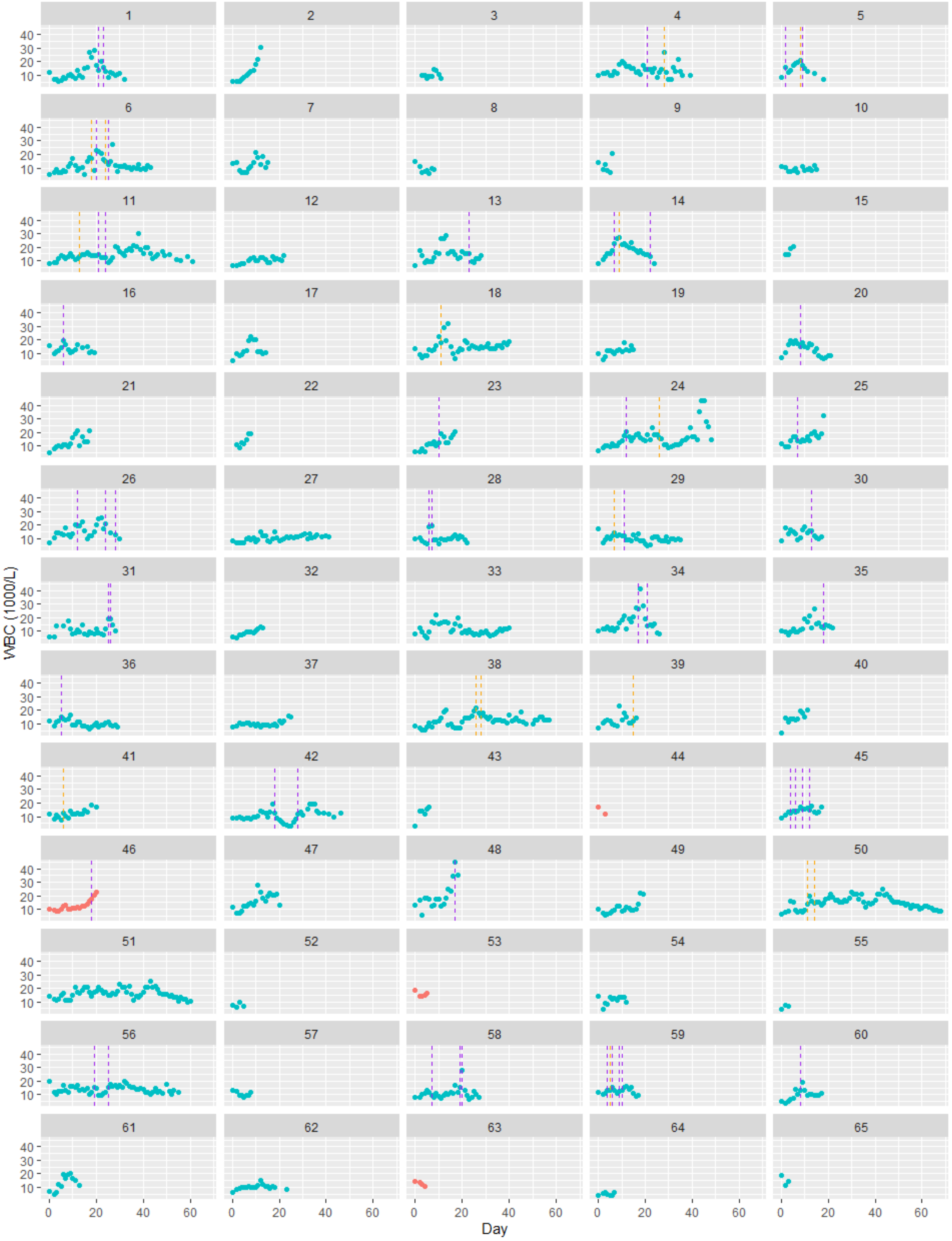

Figure S3. WBC trajectories over time for individual patients (in 1000/L). Green=WBC rise; red=no WBC rise. Purple=VAP/VAT event; orange=LCBI event.

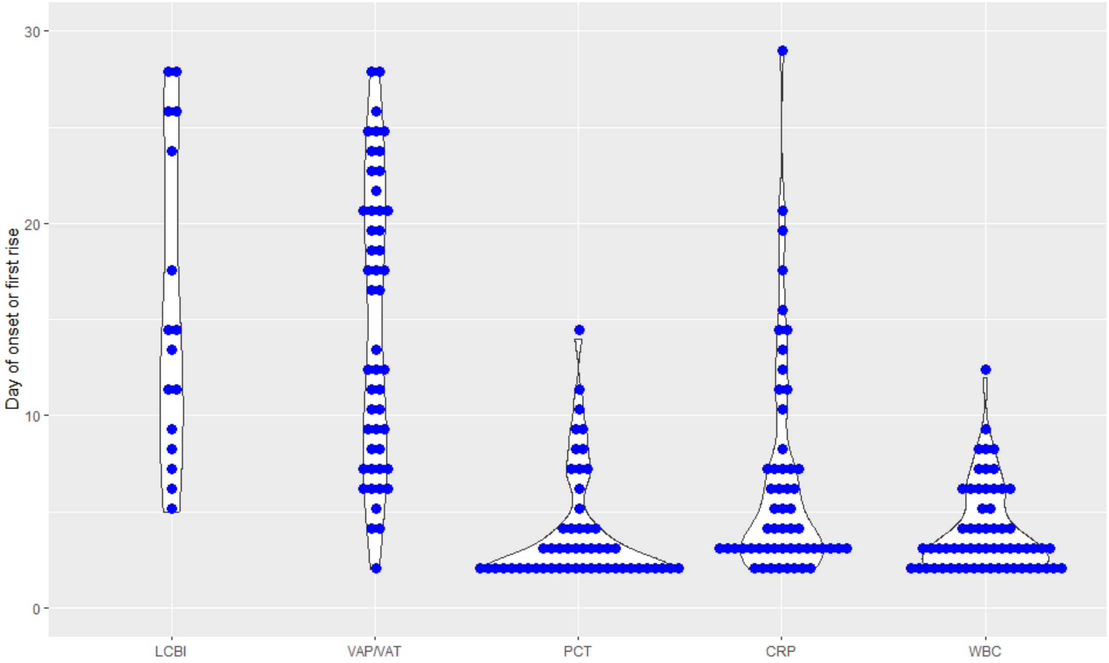

Figure S4. Violin plots for days of LCBI and VAP/VAT onset and first biomarker rise.
